# Population distribution and household conditions in Saudi Arabia: reflections from the 2010 Census

**DOI:** 10.1186/2193-1801-3-530

**Published:** 2014-09-16

**Authors:** Asharaf Abdul Salam, Ibrahim Elsegaey, Rshood Khraif, Abdullah Al-Mutairi

**Affiliations:** Center for Population Studies, King Saud University, Riyadh, Saudi Arabia

**Keywords:** Population and housing, Persons per household, Urbanization, Major regions, House construction, Basic infrastructure

## Abstract

The Kingdom of Saudi Arabia, the largest nation in the Arabian Peninsula is divided into 13 regions, which are of different development levels in terms of both population and public utility infrastructure. More than the other regions, population is high in Al-Riyadh, Makkah Al-Mokarramah, and the Eastern Region, due to urbanization. The current analysis of census results is aimed at understanding (i) regional variations in population and households and (ii) house ownership, type of housing, and housing infrastructure.

Saudi Arabia’s population is 26,090,555, living in 4,655,127 households with higher concentration in Al-Riyadh, Makkah Al-Mokarramah, and the Eastern Region. One-fourth of the households are in Makkah Al-Mokarrammah while another one-fourth in Al-Riyadh. Households are small with 6 persons in each. The proportion of households in own houses is less than half – mostly Saudi households. Households in the Kingdom live in apartments, traditional houses, villas or floor in a villa with differing proportions across regions and between Saudi and non-Saudi. While apartments are the major type of housing (major regions), traditional houses (Jazan, Al-Baha, Hail, and Aseer regions) and villas (Al-Riyadh region) still exist that are built by concrete (less than four-fifths), block/brick (less than one-fifth), mud (rare), and stone (rare) with varying regional proportions. Infrastructure – electricity, water, and sewage – vary across regions. The major source of electricity is public station followed by private station and private generators. Water source is mainly the public water inside the pipe unit but catchment tank water and well water are used. Three types of sewage system are prevalent in the Kingdom - public sewage, ditch sewage, and private sewage.

An inequitable distribution of population across regions shows urbanization, causing an emergence of the modern housing sector influencing house ownership. Developed regions have less ownership, more rentals and office quarters, thus, presuming that development level and migration status are driving house ownership. The impact of infrastructural variations is difficult to interpret as such variations affect the interest of the public sector and customers. A lesser dependence on the public sector requires caution when assessing the situation and when creating policies and provisions to improve quality of life.

## Introduction

The population of Saudi Arabia constitutes 7.4 percent of 359 million in the Arab world and 65.1 percent in the Gulf Cooperation Council -GCC (Salam and Elsegaey 2012; Rashad 2000). Even though the demographic lag – high fertility and low mortality (Freedman 1995; Jacobson 1994) – is of debate in the Arab world, in general, and specifically the ongoing fertility transition in Saudi Arabia (Khraif 2009; Courbage 1999) is receiving wider acceptance. The resultant improvement in health status promotes development by demanding increase in resources necessary for a better life (Shawky 2001); hopefully, unfolding a new demographic era (United Nations 2002).

Rapid urbanization, intensive use of water resources, consumption patterns, lifestyles, and increased industrialization in Saudi Arabia exacerbate the challenges of protecting the environment and of addressing related problems – water scarcity, pollution, solid waste, and biodiversity (UNDP 2004). Water scarcity affects not only Saudi Arabia but also the Middle East and North African region and this challenge creates conflicts among countries and population groups, adding to the political instability of the region (Fahimi and Kent 2007).

The Kingdom of Saudi Arabia’s three-fifths of the population live in major cities, which are well organized with relatively integrated transportation networks and most basic services (UNDP 2004). This alarming over-urbanization creates an increase in demand for public services such as piped water, electricity, sewage, and telephone beyond the rapid evolution of urban centers, creating a social burden (United Nations 2006; Makki 1986); and exacerbated the demand for basic necessities such as housing and transportation (Al-Gabbani 2008). The two challenges faced by urban development authorities in the Kingdom are (i) meeting the increasing demand for services due to population growth and urban expansion and (ii) enabling the private sector to play an increasing role in providing additional facilities as well as handling the operation and maintenance of existing ones (UNDP 2004).

The accelerated urbanization in Saudi Arabia (Khraif 2007) resulting from natural increases, immigration, and rural urban migration, was attributed to the inability of the rural economy to absorb its population (Makki 1986). The spatial distribution policies of Saudi Arabia have appreciated even though there are widespread disparities in physical and social infrastructure (Al-Khalifeh 1993).

Both the Saudi government and the private sector have made extensive efforts to create adequate housing in various forms - free housing schemes, land plots, and housing loans to promote ownership of housing units, among the Saudi citizens (United Nations 2006; UNDP 2004). The housing sector in the Kingdom is in debate with regard to the seismic zone and design parameters applicable in the Kingdom to protect the population from the seismic vulnerability of existing buildings (Al-Haddad and Siddiqi 1995). In addition, innovation diffusion - a trend of the housing sector in the Kingdom (Rahmaan, Rahmaan and Al-Shaye, 1990) is to be adopted with a “ready to wear” approach. Modern architecture in housing construction is also under criticism in terms of (i) the desire to preserve the traditional architectural style of Saudi cities and towns, (ii) the confusion of Islamic architecture, and (iii) the dissatisfaction with the abstraction of some modern architectural forms. The chaotic appearance of a majority of modern villas due to the overuse of ornaments and the presence of too many building materials in their facades (Al-Ibrahim 1990) are also of concern. Therefore, a need for contemporary architecture arises (Ghazzeh 1995).

Sewage, the primary source of water pollutants in Saudi Arabia, requires further expansion as a large portion of water, industrial, and petroleum wastes are not connected to sewage treatment plants (UNDP 2004). Rising to the challenge of multiple environmental pressures due to continued economic growth, the demand for energy and desalinated water is critical to counter public health risks in the Kingdom (UNDP 2004).

Overall, the alarming urbanization trend in Western Asia makes decision-making increasingly decentralized to cities and metropolitan areas, leading to an increasing demand for urban infrastructure and services such as affordable housing, water supply and sanitation, electric power, solid waste disposal, and health and education (United Nations 2006).

## Objectives

The current paper attempts at a detailed analysis of the Population and Household Census 2010 tables (final) to detail (i) regional variations in population and households and (ii) house ownership, type of housing, and housing infrastructure, namely, the materials used for construction and electricity, and water and sewage facilities. Efforts are made to delineate Saudi and non-Saudi population as they differ widely in their outlook and lifestyle.

Census operations in Saudi Arabia started in 1962–63 but have not been succeeded to establish a periodicity even though subsequent censuses were held in 1974, 1992, 2004 and 2010. Census reports differ from one another in terms of definitions, classifications and the tables. However, 2004 and 2010 censuses are comparable, where household tables have been consistently reported. Household Tables of Census 2010 offer data by regions and governorates for indicators namely type of household (traditional, villa, floor in a villa, floor in a traditional house, apartment and others); built up material (concrete, block/brick, mud, stone and others); house ownership (owned, rented, provided by employer and others); source of electricity (public station, private station, private generator, others and no data); source of water (public inside the piple unit, catchment tank, well and others) and type of sewage faciltiy (public sewage, ditch, private sewage and others). Each category has the number of housing units, number of households and number persons in it.

## Results and discussion

As per the Population and Household Census 2010’s results, Saudi Arabia has a population of 26,090,555. Three-fourths of them are Saudi natives and the rest are non-Saudi immigrants (Table 1) from various parts of the world. Immigrants hail mostly from Southeast Asia and Africa (Khraif 2009; Khraif 2000; Clarke and Murray 1973). Though the Saudi population, with the structural changes in labor force, shifted from agriculture to the service sector, the non-Saudi population remains evenly distributed (Khraif 2000). While the Saudi natives have a higher number in less urbanized regions such as Northern Borders (88.0%), Al-Baha (87.2%), and Tabouk (86.5%), non-Saudi population has a higher proportion in highly urbanized regions such as Al-Riyadh (31.7%); Makkah Al-Mokarramah (35.8%); Eastern Region (21.8%), and Al-Madinah Al-Monawarah (23.5%) (Salam and Mouselhy 2012; Khraif 2007; Sly and Serow 1993), indicating a rapid urbanization and concentration in major cities (Al-Gabbani 2008), and an unorganized movement of population towards urban centers (Makki 1986). Not only in Saudi Arabia but also elsewhere in the Gulf Cooperation Council (GCC), the number of expatriates increased, pressuring the economy and society (Khraif 2009). A large number of expatriates add to the demographic challenges of Saudi Arabia in addition to the youth bulge, high fertility, and high growth (Collymore 2003). In the region, expatriates are essentially required to develop infrastructure and power stations, government ministries and services, and industrial and agricultural sectors (Wincker 1997; Sufian 1993; United Nations 1990).

**Table 1:** **Population of Saudi Arabia by region**

Regions	Population	Percent Saudi	Percent region wise	Persons per household
Al-Riyadh	6505509	69.3	24.9	5.6
Makkah Al-Mokarramah	6662597	64.2	25.5	5.0
Al-Madina Al-Monawarah	1694749	76.5	6.5	5.5
Al-Qaseem	1184365	80.8	4.5	5.8
Eastern Region	3799773	78.2	14.6	6.1
Aseer	1897236	85.7	7.3	5.6
Tabouk	777680	86.5	3.0	5.8
Hail	593308	85.6	2.3	6.3
Northern Borders	311473	88.0	1.2	7.3
Jazan	1332262	84.2	5.1	6.6
Najran	496613	82.5	1.9	5.8
Al-Baha	406724	87.2	1.6	5.4
Al-Jouf	428266	83.7	1.6	6.1
Total	26090555	74.1	100.0	5.6

Proportionately, the Makkah Al-Mokarramah region has the Kingdom’s largest share of population (25.5%), followed by Riyadh (24.9%) and Eastern Region (14.6%); thus, totaling 65.0 percent. Such an imbalance of population has been in existence in the Kingdom on regional and urban scales (Makki 1986), which may have been due to cultural factors and the concentration of government and private spending (Al-Khalifeh 1993). These regions are highly developed, with metropolises and high levels of industrial and commercial activity (Khraif 2000; Al-Abdulkareem and Ballal 1998) – Ar-Riyad (Al-Riyad Region), Jeddah, and Makkah (Makkah Al-Mokarramah Region), and Dammam (Eastern Region). These metropolises attract not only the non-Saudi manpower from other countries but also the Saudi manpower from other regions, creating an imbalance of population in Northern Borders, Al-Jouf, Al-Baha, and Najran regions (Khraif 1994). Such urbanization trends are not only characteristic of Saudi Arabia but also of West Asia (Televizian 2009; Fahimi and Kent 2007).

Even though Al-Riyadh and Makkah Al-Mokarramah have an almost equal share of the Kingdom’s population (24.9% and 25.5%), there is an imbalance of non-Saudi share with Makkah Al-Mokarramah having 35.3 percent, but Al-Riyadh has 29.6 percent. The overall equal share in these regions has resulted from the share of the Saudi population in those regions (23.3% and 22.1%), but there is a correlation between the number of Saudi population and the number of non-Saudi population (Pearson correlation coefficient = 0.987; p = 0.000). That is, the number of non-Saudi depends upon the number of Saudi population. With an increase in the number of Saudi natives in a region, the number of non-Saudi also increases. In other words, rural to urban migration of Saudi natives leads to an increase in non-Saudi population in the urban area.

While Al-Riyadh carries the largest share of Saudi population (23.3%), Makkah Al-Mokarramah carries the largest share of non-Saudi population (35.3%), and of the total population (25.5%). The Eastern Region has 15.4 percent of the Kingdom’s Saudi population and 12.3 percent of non-Saudi population. It shows that there is population concentration in urbanized regions where three-fourths of the population live in Al-Riyadh, Makkah Al-Mokarramah, and the Eastern Region. While two-thirds in the Kingdom are Saudi population, the rest are foreigners (non-Saudi), concentrated on urbanized regions, especially in large cities such as Riyadh, Jeddah, Dammam, Makkah, Madina, and Buraydah. It is in these cities where the non-Saudi population lives and participates in commercial and service sectors. The number of Saudi and non-Saudi populations living in regions – major or others – is statistically associated (χ^2^ = 587346; P < 0.01).

The population of Saudi Arabia lives in 4,655,127 households (2,999,218 of Saudi and 1,655,909 of non-Saudi). Similar to population, household concentration can also be understood. The population per household does not vary widely across governorates, thus, making the number of households in a region as a function of number of persons. Still, some differences in the household size are observed regionally; ranging from 5.0 to 7.3 persons. The Saudi household size varies noticeably from that of non-Saudi. While the Saudi household size ranges from 5.5 to 8.4 (average of 6.4); the non-Saudi household size ranges from 2.6 to 4.3 (average of 4.1). Thus, the Saudi households are larger than non-Saudi households by 2 persons, which may be attributed to the difference in their residential status. While the Saudi population is composed of permanent residents, the non-Saudi population is composed of temporary residents. A vast majority of non-Saudi population in the Kingdom live alone, keeping their families in their native homes.

### House ownership

**Figure 1:**
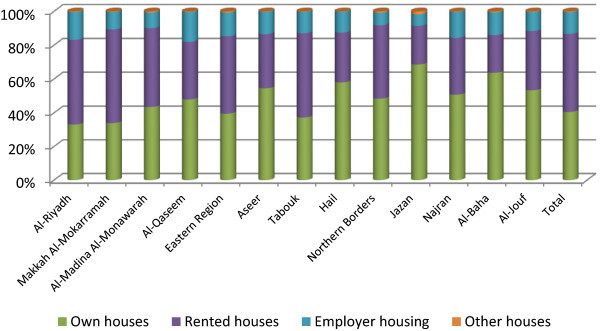
**House ownership pattern in the Kingdom.**

Housing supply seldom equates to demand. Even though there is a surplus of housing – with medium and high cost - most people cannot afford to buy or rent a proper house (Wahab 1991) and so, solving the housing problems needs concerted efforts of private agencies. House ownership does not appear high in the Kingdom due to intense rural to urban migration and immigration. While 40.5 percent of households have owned houses (Figure 1), 46.2 percent have rented and 12.8 percent have employer housing. The Jazan region has a large share of households at own houses (68.6%); Al-Riyadh and Makkah Al-Mokarramah regions have the lowest shares (33.1% and 33.9%); and Al-Baha (63.6%) has a higher share of owned houses. The existing house ownership pattern of Saudi families is a positive outcome of the government’s housing policies (UNDP 2004). The mean number of households at own houses varies widely between major regions and others (359,281 as against 80,679: t = 6.39; p < 0.01). That is, the number of owned houses differs from region to region; major regions have more owned houses but are affected by the number of households in the region.

Three-fifths of Saudi households are in owned houses (60.0%); nearly two-fifths are in rented (34.8%), and a few in employer housing (4.5%). Despite the high per-capita income of the Saudi population in the Kingdom (UNDP 2004), house ownership remains low, attributed to frequent migrations and temporary settlements. Major inmigrating regions – Al-Riyadh, Makkah Al-Mokkarrammah, and the Eastern Region – face such challenges whereas Al-Baha (85.4%), Jazan (85.3%), Hail (78.6%), and Al-Jouf (74.7%) have comparatively higher proportion of Saudi households at own houses. There is a positive correlation between the number of households and the number of owned houses in the Kingdom (Pearson correlation = 0.985; P <0.01). That is, the owned houses increase with increase in the number of households in a region. On the contrary, the Tabouk (46.6%) region has a low proportion where there are house-renting supplements, depending upon the availability of suitable employer housing. Houses rented by Saudi households are highest in Makkah Al-Mokarramah (40.7%), Al-Riyadh (39.8%), the Eastern Region (36.7%), and Al-Madina Al-Monawarah (36.1%). Rented houses are preferred over employer housing in the Kingdom because of (i) locational advantages, (ii) physical and spatial requirements, and (iii) affordability. Office quarters are used more in the Eastern Region (7.3%), Tabouk (6.6%), and Al-Riyadh (6.2%).

The preference for independent and hassle- free accommodation away from official commitments creates a demand for rented housing. Non-availability of official accommodation in the private sector may also influence the decision to rent houses (Al-Jassem 2013). The continually increasing demand for real estate, the lag in supply, and increasing prices are hurdles for potential buyers.

Wherever there is an employer housing, house rental decreases, for example, Al-Qaseem has newly developed public academic and medical institutions with in-house residential facilities that reduced house-renting behavior. This is a crisis that house ownership has no pattern in the country. Accommodation is part of employment contract in the Kingdom; all employees receive houses or house rent allowance (HRA). Reporting of rental houses appears higher due to the inclusion of the latter. It was supposed to be categorized into employer housing.

Housing requirements as well as housing arrangements of Saudi natives vary from that of non-Saudi residents. Office quarters offer accommodation to non-Saudi more often in Al-Qaseem (48.8%), Al-Baha (48.7%), Aseer (42.4%), and Hail (41.6%) regions. Other types of households are found in Jazan (3.5%), more often than in other regions. Office quarters are highest in Riyadh (31.9%), followed by Makkah Al-Mokarramah (22.1%), and the Eastern Region (11.9%).

House-renting conditions are severe in urbanized regions - Al-Riyadh, Makkah Al-Mokarramah, and the Eastern Region. Tabouk has a higher proportion of households in rented housings, where employer housings are low: a situation of housing policies in the region. The lack of housing facilities by employers creates hurdles in accommodation that cause a boom in the real-estate sector in the country, catering to renters (Table 2).

**Table 2:** **Differential house ownership and renting pattern of Saudi and Non Saudi population across regions**

	Saudi				Total	Non Saudi			
	Own houses	Rented houses	Office quarter	Other houses		Own houses	Rented houses	Office quarter	Other houses
Al-Riyadh	53.5	39.8	6.2	0.5	100.0	3.8	65.0	31.0	0.2
Makkah Al-Mokarramah	54.3	40.7	4.5	0.5	100.0	5.6	75.7	18.3	0.3
Al-Madina Al-Monawarah	61.1	36.1	2.1	0.6	100.0	5.0	69.4	24.8	0.9
Al-Qaseem	70.0	27.3	2.1	0.6	100.0	3.7	47.3	48.8	0.1
Eastern Region	55.0	36.7	7.3	1.1	100.0	5.5	65.9	28.5	0.1
Aseer	71.8	24.7	3.2	0.4	100.0	3.7	53.7	42.4	0.2
Tabouk	46.6	46.4	6.6	0.3	100.0	4.7	61.9	33.2	0.2
Hail	78.6	20.2	0.9	0.3	100.0	4.9	53.3	41.6	0.1
Northern Borders	61.9	35.2	1.8	1.0	100.0	4.6	69.6	25.7	0.1
Jazan	85.3	12.3	1.1	1.3	100.0	16.6	55.8	24.1	3.5
Najran	70.6	26.0	3.0	0.3	100.0	6.1	50.9	42.6	0.4
Al-Baha	85.4	12.9	0.8	0.9	100.0	2.4	48.8	48.7	0.1
Al-Jouf	74.7	23.5	1.4	0.4	100.0	3.0	62.3	34.3	0.3
Total	60.0	34.8	4.5	0.6	100.0	5.1	66.7	27.8	0.4

Makkah Al-Mokarramah has the highest share of total households owning their houses (23.9%), followed by Al-Riyadh (20.3%), Eastern Region (13.0%), Jazan (7.3%), and Al-Madina Al-Monawarah (7.1%) regions. As this has a relation with the total households in the region, the position of Jazan in house ownership may be appreciated. Being a region with the highest percent of households in own houses, the region’s housing policies are appreciated. With employer housing and rented housing hardly found, the region sets an ideal situation. Similarly, Al-Baha region has 63.6 percent of owned housings and 13.4 percent of employer housings; together, they create a low proportion of rented houses.

There are differences between Saudi and nonSaudi households in terms of house ownerships. The right to own a house is offered to Saudi natives even though the real-estate sector has also started opening up to non-Saudis. The non-Saudi Arab population in certain localities has already secured their right as house owners. Still, their proportions remain at 5.1 percent. Jazan has encouraged such occupations as 16.6 percent of the region’s non-Saudi households have their own houses.

The employment sector drives house ownership in the Kingdom. Levels of deficit exists in the local workforce across labor groups and in various locations. In addition, the female workforce penetrating foreign labor in the future is expected to affect house ownership (Khraif 2000). All these influence real-estate development and house ownership pattern in the Kingdom.

Sixty percent of Saudi households in the Kingdom own their houses. This low proportion of owned houses are due to rural to urban migrations or employment-oriented short-term settlements in major cities such as Riyadh, Jeddah, Dammam, Makkah, Madina, and Alkhubar. There are also upcoming industrial, academic, medical, and military townships that attract native manpower from various parts of the Kingdom, which create a boom of rented houses due to the shortage of employer housings. Such settlements exist in large number in Jubail (Eastern Region), Burayda (Al-Qaseem region), Najran (Najran region), and Tabuk (Tabouk region). As a result, the proportion of rented housing increases, unless large-scale employer housings are made. It also has implications on labor migrations and regional development initiatives. A large majority of non-Saudi households are in rented houses (66.7%) and office quarters (27.8%).

The government’s human settlement policies, focusing on the consolidation of existing settlement structure and continued efforts to improve standard of life in rural areas, changed the housing sector in the Kingdom (UNDP 2004). An artful construction of buildings at Al-Alkhalaf settlement in Aseer region is an example of integrating history of traditional buildings and village development to create technical, functional, and environment-friendly quality houses molded into modern standards, amenities, safety and permanence through vernacular architecture (Ghazzeh 1995). Supporting middle and low-income citizens to secure their own housing; ensuring adequate resources to meet the growing demand; overcoming constraints of construction costs; and accurate assessment of housing requirements are issues demanding attention (UNDP 2004).

### Types of houses and construction materials

Not only modernization and technology, but also environmental conditions affect the housing sector in a society. Saudi Arabia has its own criteria on housing construction, namely, seismic design (Al-Haddad and Siddiqi 1995); sustainable housing (Susilawati and Al-Surf 2011); environmentally suitable Islamic architecture (Al-Ibrahim 1990; El-Hamid and El-Din 1991; Ghazzeh 1995); climate and culture (Rahmaan et al. 1990); and national, regional, and local development policies (Benna 1996).

**Figure 2:**
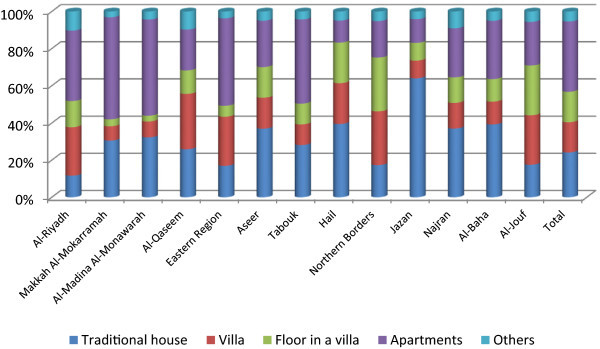
**Distribution of households by the type of house across regions.**

The highest proportion of households in the Kingdom are in apartments (41.1%), followed by traditional house (26.2%), villa (17.7%), and floor of a villa (17.7%). Makkah Al-Mokarammah has 54.1 percent; Al-Madina Al-Monawarah has 51.1 percent; the Eastern Region has 46.4 percent; and Tabouk has 46.4 percent of households in apartments (Figure 2). The Jazan and Hail regions have a low share of such housings (13.4% and 14.1%, respectively) revealing that housing developments differ regionally in the Kingdom. While major regions such as Al-Riyadh, Makkah Al-Mokarramah and the Eastern Region have developed modernized apartments, other regions have not. Rapid urbanization in the Kingdom accompanied by modernized lifestyle prompt lot owners and investors to create vertical development in the housing sector beyond sustainable interventions, thus, blocking solar energy, natural light, and ventilation (Susilawati and Al-Surf 2011), which will have far reaching negative impacts on population and health. Thus, single family house incorporating the privacy features and greater number of rooms, providing for segregation of sexes and the guests appears to be the preferred type of dwelling unit in the Kingdom (Rahmaan et al. 1990).

While traditional houses are on a decline in Al-Riyadh (11.5%), the Eastern Region (16.7%), and Northern Borders (20.2%), regions such as Jazan and Hail hold such housings in larger proportions (67.0% and 46.9%, respectively). Villa is common as an independent spacious accommodation arrangement, especially for large - joint – families; proportionately higher in Northern Borders (33.7%), Al-Jouf (33.3%), and Al-Qaseem (29.3%) regions, where the floor of a villa is also used equally. Such housings overcome issues of residents’ privacy, which is a major reason for interpersonal conflicts in the cultural context (Susilawati and Al-Surf 2011). A dual phenomenon in housing characterized by (i) adoption of villa and its allied innovations and (ii) revival of Arab style of house and the adaption of villa into a courtyard-type dwelling unit (Rahmaan et al. 1990) is preferred. House ownership has a positive correlation with housing type that with increasing ownership, apartments increase faster (Pearson correlation = 0.906; p < 0.01), than traditional house (Pearson correlation = 0.843; p < 0.01) or villa (Pearson correlation = 0.772; p > 0.01), in the case of Saudi households.

**Figure 3:**
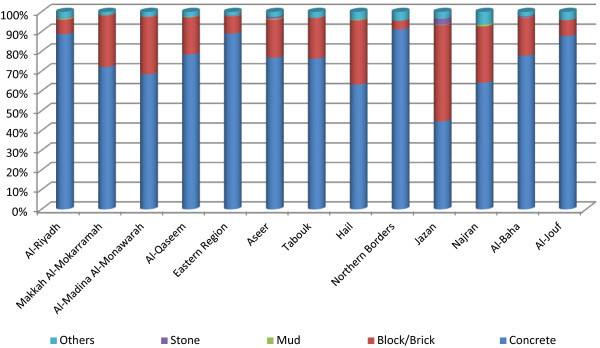
**Regional distribution of households by the material used for their house construction.**

Similar situation exists in the case of materials used for housing construction. A large majority of houses in the Kingdom are concrete-built (78.1%), mainly in the three major regions, together forming 69.9 percent (Figure 3). In addition, Northern Borders and Al-Jouf regions have the majority of their households in concrete houses (91.1% and 87.8%, respectively). The Jazan region has a low proportion of concrete houses (44.7%). The Hail and Najran regions have less than two-thirds of their houses concreted where the rest live in non-concreted - block/brick - houses: an issue of housing development. Housing construction depends largely upon the environmental conditions, considering the impact of housing design on ecological setting where earth-sheltered housing schemes play an important role (El-Hamid and El-Din 1991).

Villa is a Saudi accommodation whereas other types (pota cabins, tents, and barracks) are of non-Saudi accommodation (Table 3). One-third of Saudi households are in apartments (34.3%); the rest are in traditional houses (26.9%), villa (25.5%) or floor in a villa (10.9%). For non-Saudi, two-fourths live in apartments (53.4%) and one-fourth in traditional houses (24.9%). More than one-half of the Saudi households are in apartments in Makkah Al-Mokarramah (51.8%); nearly one-half in Al-Madina Al-Monawarrah (48.9%) and Tabouk (47.8%), but only slightly more than one-third in the Eastern Region (37.4%). While the Riyadh region has 25.9 percent of Saudi households in apartments, the Najran region has 27.2 percent. The Al Baha (25.7%) and Al Jouf (22.9%) regions also have Saudi households in apartments. Traditional houses are common in Jazan (68.6%) as accommodation of Saudi households, followed by Hail (47.4%), Aseer (39.8%), Najran (35.4%), and Al-Madina Al-Monawarah (33.7%). Modern architecture has been criticized as it is environmentally unsuitable for the hot arid Saudi Arabia. Constructions of modern buildings are regulated with the aims (i) to preserve traditional architectural styles, (ii) to incorporate Islamic architecture, and (ii) to discourage abstraction of architecture of facades (Al-Ibrahim 1990).

**Table 3:** **Type of houses and construction material used**

Region	Type of house					Total	Built in material				
	Traditional house	Villa	Floor in a villa	Apartments	Others		Concrete	Block/brick	Mud	Stone	Others
Saudi											
Al-Riyadh	11.0	40.6	20.7	25.9	1.2	100.0	94.9	3.9	0.3	0.1	0.8
Makkah Al-Mokarramah	29.5	11.4	5.6	51.8	0.7	100.0	75.6	23.8	0.0	0.1	0.5
Al-Madina Al-Monawarah	33.7	11.2	4.1	48.9	1.5	100.0	69.7	28.7	0.1	0.1	1.4
Al-Qaseem	25.5	42.6	17.0	12.8	1.1	100.0	85.2	13.7	0.1	0.0	1.0
Eastern Region	18.0	35.1	7.4	37.4	0.8	100.0	91.3	7.7	0.1	0.4	0.5
Aseer	39.8	22.3	13.4	20.7	2.0	100.0	81.0	16.4	0.4	0.9	1.3
Tabouk	25.0	14.0	9.5	47.8	2.4	100.0	81.8	15.9	0.1	0.1	2.2
Hail	47.4	34.3	7.3	7.7	2.9	100.0	68.6	28.1	0.3	0.0	3.0
Northern Borders	19.8	41.9	17.7	15.9	1.8	100.0	95.3	3.0	0.0	0.0	1.8
Jazan	68.6	12.0	4.8	11.6	1.5	100.0	48.6	46.9	0.1	3.6	0.8
Najran	35.4	19.9	10.8	27.2	5.0	100.0	71.3	22.8	0.5	0.0	5.4
Al-Baha	41.5	15.8	11.6	25.7	1.1	100.0	81.6	81.6	16.9	0.0	0.7
Al-Jouf	18.4	45.9	8.5	22.9	2.5	100.0	93.6	4.3	4.3	0.0	2.0
Total	26.9	25.5	10.9	34.3	1.3	100.0	81.9	16.6	0.2	0.4	1.0
Non Saudi											
Al-Riyadh	12.3	4.7	3.9	54.5	23.1	100.0	80.2	11.8	1.3	0.3	6.4
Makkah Al-Mokarramah	30.8	2.4	0.9	57.4	6.6	100.0	67.6	29.1	0.1	0.1	3.0
Al-Madina Al-Monawarah	27.9	2.5	1.0	55.9	10.6	100.0	66.0	29.0	0.3	0.6	4.1
Al-Qaseem	25.5	2.7	3.3	39.5	26.7	100.0	64.9	28.9	0.4	0.1	5.7
Eastern Region	13.6	6.4	2.2	66.4	9.9	100.0	84.3	10.8	0.3	0.1	4.5
Aseer	36.4	3.0	1.6	42.8	14.7	100.0	64.1	27.6	0.7	1.4	6.2
Tabouk	41.8	2.2	1.7	41.5	11.2	100.0	57.1	36.5	0.3	0.4	5.7
Hail	45.6	4.8	3.1	30.6	13.3	100.0	49.5	43.6	0.6	0.1	6.2
Northern Borders	21.4	7.2	3.1	45.5	19.7	100.0	77.4	8.9	0.1	0.1	13.5
Jazan	62.1	3.7	1.0	19.0	13.1	100.0	32.5	54.2	0.4	1.3	11.6
Najran	46.8	2.2	1.6	29.2	19.5	100.0	48.2	41.3	1.7	0.1	8.7
Al-Baha	30.4	1.3	6.2	45.5	15.8	100.0	67.4	25.5	0.1	0.6	6.4
Al-Jouf	30.2	3.8	1.3	44.5	17.6	100.0	74.1	16.6	0.1	0.1	9.0
Total	24.9	3.7	2.2	53.4	14.1	100.0	71.1	22.9	0.6	0.3	5.1

Regional differences in types of housing have a bearing on climatic conditions and also on livelihood. For example, the Northern Borders region has a mix of traditional houses and villas for Saudi households but apartments and traditional houses for non-Saudi households.

A large majority of Saudi households are in concrete housings (81.9%), followed by block/brick houses (16.6%) whereas comparatively more non-Saudi households are in non-concrete houses in the Kingdom. Houses made of mud (0.2%) or stone (0.4%) are rare but more among non-Saudi households. Houses of other built are often used by non-Saudi, more often in Northern Borders (13.5%) and Jazan (11.6%). Nearly all Saudi households in Northern Borders (95.3%), Al-Riyadh (94.9%), and Al Jouf (93.6%) live in concrete houses. However, only two-thirds of their non-Saudi counterparts of these regions live in such houses. Concrete housings of Saudi and non-Saudi are less in Jazan (48.6%, 32.5%), Al-Madina Al-Monawarah (69.7%; 66.0%), and Najran (71.3%; 48.2%). Modernization brings imported western design models admired for appearance, but ignoring complexity of natural environment in the area (Ghazzeh 1995).

### Household infrastructure

Infrastructure – electricity, water and sewage facilities are less dependent on the public sector, even though there is growth in electricity generation and water desalination capacity (UNDP 2004), reflecting an expanding private sector in the Kingdom. Demand for services is increasing due to population growth and urban expansion along with the need to expand services, thus, enabling the private sector to offer additional facilities. Guidelines pertaining to air and water quality, hazardous wastes, occupational health, and noise require effective monitoring of compliance to ensure prevention of environmental hazards (Benna 1996), as in Jubail Industrial city – an example of sustainable water and sanitation program.

**Figure 4:**
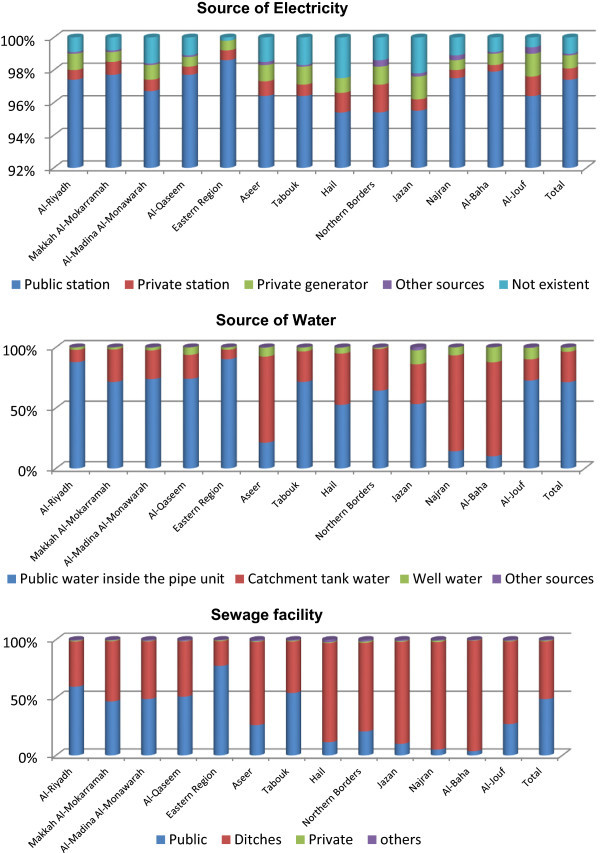
**Distribution of households by housing infrastructure at households by regions.**

A large majority of total households depend upon public station for electricity (97.4%); all regions having above 95.0 percent (Figure 4). Hail, Northern Borders, Jazan, and Al-Jouf have lesser dependence where private generator supplements (95.3%, 95.4%, 95.5%, 96.3%, respectively). Rural to urban migrations and immigrations to major urban centers raise the demands for public services - piped water, electricity, sewage, and telephone (Makki 1986). The power consumption pattern has a close relation with the building envelope, as exemplified by the one-storey houses in Dhahran City (Al-Mofeez 2007). The type of electricity used in a Saudi household is correlated with the house ownership more strongly positive with public station (Pearson correlation = 0.992; p < 0.01), private station (Pearson correlation = 0.966; p < 0.01), and private generator (Pearson correlation = 0.792; p < 0.01), in this order.

Both Saudi and –non-Saudi households depend upon public station for electricity even though with differentials. However, Northern Borders, Al-Jouf, and Jazan regions show higher differentials (9.5, 7.4, and 4.7 points, respectively), where non-Saudi depends upon private generators (Al-Jouf) or other sources (Northern Borders and Jazan). Aseer, Jazan, and Najran have even Saudi households depending upon private generators and other sources, to an extent. The energy requirements in a house may well be addressed by passive solar energy (El-Hamid and El-Din 1991) – a step toward low energy costs and a functionally inscribed sustainable natural social environment.

Water supply in Saudi Arabia receives both appreciation and criticisms (Khraif 2009) even though mineral concentration has been proved at par with the Saudi Arabian and international standards (Hashem and Al-Johany 1994). A sustainable water resources management plan considering water quality, flood management, perennial flow, water balance and utilization of recycled water was proposed to meet the requirements in the Riyadh region through Wadi Hanifa (Alhamid et al. 2007). Drinking water, sanitation, hygiene and water-resource management strategies support the nation’s goal of reducing burden of disease, and this is where Saudi Arabia receives an appreciable position (Pruss-Ustun et al. 2008).

Sources of water in the Kingdom are divided into public water inside pipe units, catchment tank water, and well water. There are other sources as well. Public water facilities are almost universal but only 71.3 percent of households have access. Catchment tank water serves 25.0 percent of households; well water to 3.4 percent and other sources to 0.3 percent. However, public water facility suffers in Al-Baha (10.0%), Najran (14.2%), Aseer (21.3%), and Hail (52.5%) regions. Water requirements in these regions are supplemented by catchment tank. However, such arrangements are even insufficient in Jazan and Al-Baha, where households have wells (11.6% and 12.3%, respectively). The house ownership is a determining factor of source of water as there is a positive correlation between house ownership and public water inside pipe units (Pearson correlation = 0.936; p < 0.01), catchment tank water (Pearson correlation = 0.707; p < 0.01), and well water (Pearson correlation = 0.498; p < 0.10).

Desalination plants provide water to households in Saudi Arabia, which is substituted by authorities’ wells and farm wells (Hashem and Al-Johany 1994; US- Saudi Arabian Business Council 2009). The restructuring and privatization of the Kingdom’s water sector through the establishment of a public–private partnership have received attention, and this is instrumental in developing the industry and delivering water and wastewater-related services to residents throughout the country (US- Saudi Arabian Business Council 2009).

Saudi households depend upon public water inside pipe units (69.3%), less than that of non-Saudi (75.0%) in Makkah Al-Mokarramah (67.1% as against 77.4%); Al-Madina Al-Monawarah (70.7% as against 80.6%); Aseer (19.7% as against 26.3%); Hail (50.8% as against 56.9%); Northern Borders (61.9% as against 72.2%), and Jazan (52.9% as against 54.5%) region (Table 4). Saudi households in these regions depend upon catchment tank water as well, except in Jazan, with well water use. The Saudi population in most of the regions, more often than non-Saudi, depends upon catchment tank water. Dependence on well water by Saudi households appears highest in Al-Baha (12.7%). Not only the quality of water but also the pipelines or the mode of supply affect the potability of water (El-Rehaili and Misbahuddin 1995).

**Table 4:** **Distribution of household by housing infrastructure**

Region	Electricity				Water			Sewage		
	Public station	Private station	Private generator	Others	Public	Catchment tank	Well + others	Public	Ditches	Private + others
Saudi										
Al-Riyadh	98.7	0.4	0.3	0.6	89.2	10.0	0.8	55.7	43.3	1.0
Makkah Al-Mokarramah	98.4	0.6	0.4	0.7	67.1	30.6	2.2	40.4	58.5	1.1
Al-Madina Al-Monawarah	96.9	0.6	0.9	1.6	70.7	27.6	1.8	44.2	54.3	1.5
Al-Qaseem	98.6	0.3	0.3	0.8	75.7	21.4	3.0	50.2	48.4	1.4
Eastern Region	99.3	0.4	0.2	0.1	91.4	7.4	1.2	77.6	22.0	0.4
Aseer	96.8	0.8	1.0	1.5	19.7	72.4	7.9	24.6	74.0	1.4
Tabouk	97.2	0.6	0.5	1.7	71.8	26.6	1.6	55.5	43.3	1.2
Hail	96.6	0.5	0.3	2.6	50.8	46.6	2.5	12.0	85.3	2.8
Northern Borders	97.7	1.3	0.7	0.3	61.9	37.9	0.3	17.1	82.0	0.9
Jazan	96.6	0.7	1.5	1.3	52.9	32.5	14.7	10.2	88.7	1.1
Najran	97.0	0.6	0.6	1.8	14.8	79.5	5.6	5.8	91.6	2.6
Al-Baha	98.3	0.4	0.4	0.5	11.0	76.2	12.7	3.9	95.5	0.6
Al-Jouf	98.5	0.4	0.4	0.8	73.4	19.6	7.0	25.5	73.2	1.3
Total	98.2	0.5	0.5	0.8	69.3	27.5	3.2	45.1	53.8	1.1
Non Saudi										
Al-Riyadh	95.6	0.7	2.1	1.5	85.7	10.2	4.1	64.8	32.7	2.5
Makkah Al-Mokarramah	96.7	1.1	0.9	1.3	77.4	21.3	1.3	55.5	42.7	1.8
Al-Madina Al-Monawarah	96.4	0.9	0.9	1.8	80.6	15.1	4.3	58.7	39.5	1.7
Al-Qaseem	96.1	0.7	1.1	2.1	70.5	16.4	13.2	52.0	45.5	2.4
Eastern Region	96.9	1.1	1.4	0.6	87.1	8.6	4.3	76.8	19.8	3.4
Aseer	95.7	1.3	0.9	2.2	26.3	67.0	6.8	30.7	65.9	3.5
Tabouk	93.8	1.1	3.1	2.0	70.6	19.9	9.4	49.4	47.7	2.9
Hail	92.1	3.1	2.5	2.4	56.9	30.8	12.3	9.6	87.4	3.0
Northern Borders	88.2	3.3	2.4	6.2	72.2	22.9	4.9	32.3	59.0	8.6
Jazan	91.9	1.0	1.4	5.8	54.5	32.8	12.8	9.6	85.9	10.0
Najran	98.7	0.5	0.4	0.5	12.8	78.2	9.0	3.2	95.8	1.0
Al-Baha	96.9	0.5	1.1	1.6	7.2	81.6	11.2	2.7	95.6	1.6
Al-Jouf	91.1	3.0	4.0	1.8	70.2	12.4	17.4	31.0	67.0	2.0
Total	96.0	1.0	1.4	1.5	75.0	20.4	4.6	55.3	42.3	2.5

Only 48.7 percent of households depend upon public sewage system but 49.7 percent depend upon ditch sewage. Private and other sewage users are rare in the Kingdom (forming 2.5%). There is poor coverage of public sewages in Al-Baha (3.6%), Najran (5.0%), Jazan (10.0%), and Hail (11.3%) regions. Northern Borders (20.7%) and Aseer (26.1%) have less than one-third of households depending upon public sewage, where a majority depend upon ditch sewages. At the same time, public sewages are used extensively in the Eastern Region (77.4%), Al-Riyadh (59.5%), and Al-Qaseem (50.8%) regions. House ownership is correlated with the type of sewage system: public sewage (Pearson correlation = 0.908; p < 0.01); ditch sewage (Pearson correlation = 0.937; p < 0.01); and private sewage (Pearson correlation = 0.942; p < 0.01).

A large majority of Saudi households use ditch sewage (53.8%), followed by public sewage (45.1%), but the reverse is true in the case of non-Saudi households (42.3% as against 55.3%). Saudi households rarely use private and other sewages as compared with non-Saudi households. Saudi households depend upon public sewage more often in the Eastern Region (77.6%); Al-Riyadh (55.7%), and Tabouk (55.5%). Dependence on ditch sewage remains high in Al-Baha (95.5%), Najran (91.6%), Jazan (88.7%), and Hail (85.3%). Private sewages are found more in Najran and other sewages in Hail, Al-Madina Al-Monawarah, and Najran. With the increasing number of residential, industrial, and business units, there is an increasing requirement for the capacity of sewage and drainage networks in order to handle not only effluents but also to treat sewages collected from septic tanks and other sources (UNDP 2004).

## Conclusions and recommendations

The “Population and Housing – Census 2010” offers valuable data on the population of Saudi Arabia and the housing arrangements, facilitating regional comparisons.

An inequitable distribution of population across regions shows an urbanization trend from rural to urban migration. Riyadh, Makkah Al-Mokarramah, and the Eastern Region grew in terms of modernized residential pockets equipped with quality living arrangements and infrastructure. Madina Al-Monwarah, Al-Qaseem, and Tabouk are also emerging in terms of modern residences, whereas the other regions are yet to set their standards. Still, the regional headquarters – municipal cities – have modernized housing and infrastructure. Overall, the housing sector is driven or influenced not only by development, but also by geographical region, climatic condition, environmental considerations, and cultural requirements.

While the major regions have low levels of house ownerships among the Saudi households, other regions have higher ownership levels, depending upon the degree of urbanization and modernization. Thus, house ownership is part of lifestyle determined by the development level of the place of residence. With the increasing development level, in-migration and immigration increase, leading to floating population reducing house ownership.

Power, water, and sewage - the basic infrastructure requirements - are expected to be provided by the public sector in any society. However, it depends upon the population’s capability to afford independent arrangements. The households in the Kingdom depend partially upon the public sector for such basic needs. Water supplied by the public sector is substituted by catchment tanks and wells, depending upon the availability of such sources even though potable water is mainly the privately supplied bottled water. Sewage system suffers seriously in the Kingdom.

There are differences between Saudi and non-Saudi households in terms of living arrangements and lifestyles. While a large majority of Saudi households live in modernized housings, non-Saudi households live in houses of less quality. On the contrary, most of the non-Saudi live in urban regions within the well-connected networking. The Saudi population lives in rural areas where traditional living and livelihood predominates.

In summary, it is timely to concentrate on policies on a national perspective with an overall national development integrating all sectors of the population – housing and infrastructure. Provisions to improve water is a necessity whether piped water, catchment tank water or well water. Hygienic surroundings including sanitation and sewage be of priority.
